# Plasma Pentraxin 3 Levels Do Not Predict Coronary Events but Reflect Metabolic Disorders in Patients with Coronary Artery Disease in the CARE Trial

**DOI:** 10.1371/journal.pone.0094073

**Published:** 2014-04-04

**Authors:** Tetsuro Miyazaki, Stephanie Chiuve, Frank M. Sacks, Paul M. Ridker, Peter Libby, Masanori Aikawa

**Affiliations:** 1 Department of Medicine, Brigham and Women's Hospital, Harvard Medical School, Boston, Massachusetts, United States of America; 2 Department of Nutrition, Harvard School of Public Health, Boston, Massachusetts, United States of America; 3 Center for Cardiovascular Disease Prevention, Brigham and Women's Hospital, Harvard Medical School, Boston, Massachusetts, United States of America; Albert Einstein College of Medicine, United States of America

## Abstract

Chronic inflammation closely associates with obesity, metabolic syndrome, diabetes mellitus, and atherosclerosis. Evidence indicates that the immunomodulator pentraxin 3 (PTX3) may serve as a biomarker of these cardiometabolic disorders, but whether PTX3 predicts cardiovascular complications is unknown. We examined the association of plasma PTX3 levels with recurrent coronary events via a prospective, nested, case-control design in the CARE trial. Among 4159 patients who had a prior myocardial infarction 3 to 20 months before enrollment and also had total cholesterol levels <240 mg/dL and LDL cholesterol levels between 115 and 175 mg/dL, we measured plasma PTX3 levels at baseline by high-sensitivity ELISA in 413 cases with recurrent myocardial infarction or coronary death during a 5-year follow-up period, and in 366 sex- and age-matched controls. Cases with recurrent coronary events and controls had similar PTX3 levels, and PTX3 did not predict recurrent coronary events — a finding that contrasts with that of C-reactive protein (CRP) and serum amyloid A (SAA) in this cohort. We then associated PTX3 levels with metabolic disorders. Low plasma PTX3 levels correlated with high body-mass index, waist circumference, and triglycerides; and with low HDL cholesterol. Overall, PTX3 levels correlated inversely with the number of metabolic syndrome components. PTX3 levels also correlated inversely with apoCIII and tissue plasminogen activator, but did not associate with CRP. Although the study further links low PTX3 levels with various features associated with metabolic syndrome, the results do not indicate that PTX3 can predict recurrent coronary events among MI survivors.

## Introduction

Pentraxin 3 (PTX3), a member of a superfamily of acute-phase reactants highly conserved during evolution and characterized by a cyclic multimeric structure and a “pentraxin domain” in their carboxy-terminus, figures crucially in innate immunity [1], [2]. In response to inflammatory signals, the liver produces the classic short pentraxin C-reactive protein (CRP) [3], which strongly associates with cardiometabolic disorders such as obesity, metabolic syndrome, diabetes mellitus, and atherosclerosis [4]. In contrast, a variety of immune and resident cells involved in cardiometabolic disorders — including macrophages, dendritic cells, fibroblasts, smooth muscle cells, endothelial cells, and adipocytes — secrete the long pentraxin PTX3 [1]. While some previous clinical and preclinical studies report pro-inflammatory properties of PTX3, others show its anti-inflammatory actions [5]–[17]. Thus, the role of PTX3 in the pathogenesis of obesity, metabolic syndrome, diabetes mellitus, and atherosclerosis remains uncertain.

Evidence suggests that PTX3 serves as a biomarker of inflammatory diseases, including atherosclerosis and metabolic disorders [18], [19]. Yamasaki et al. reported that PTX3 levels were inversely associated with the features of metabolic syndrome in a healthy population [20]. But the intriguing possibility that PTX3 is a risk predictor of future cardiovascular complications (e.g., acute myocardial infarction) remains obscure. Other unresolved issues include which metabolic components mainly associate with plasma PTX3 levels; whether plasma PTX3 levels correlate with insulin resistance or diabetes mellitus; and the association of PTX3 with other inflammatory markers. The present study examined plasma PTX3 levels in participants of the Cholesterol and Recurrent Events (CARE) trial (UMIN-CTR; UMIN000012519) to test the hypothesis that plasma PTX3 levels predict future coronary events and associate with features typical of inflammatory cardiometabolic disorders.

## Methods

### Study population

We conducted a prospective, nested, case-control study within the CARE trial (**See methods S1**). This randomized, double-blind, placebo-controlled trial included 4159 patients with a prior myocardial infarction (MI) that occurred 3 to 20 month before enrollment. The subjects had low-density lipoprotein (LDL) cholesterol levels (total cholesterol levels <240 mg/dL and LDL cholesterol levels between 115 and 175 mg/dL). Patients underwent random allocation to 40 mg pravastatin or placebo for a median of 5 years [21]. The institutional review board at each participating clinical center approved the study, and individuals gave written informed consent [22]. During the trial, 486 patients experienced the primary endpoint of coronary death or MI. Sufficient plasma for analysis was available from 413 of these cases. We randomly selected control subjects from patients who did not have a primary endpoint, matched to the cases by sex and decade of age. Measurement of PTX3 levels of these blood samples from the CARE trial and this analysis were approved by the committee for the Protection Against Research Risks of Brigham and Women's Hospital, Harvard medical school.

### Blood collection and laboratory measurements

A fasting blood sample was taken from each patient at baseline. Serial measurements of PTX3 were performed using a high-sensitivity ELISA (Perseus Proteomics Inc., Tokyo, Japan). Plasma lipid profiles, fasting blood glucose and insulin, serum creatinine levels, and tissue plasminogen activator (tPA) were measured as previously described. High-sensitivity assays for CRP and serum amyloid A (SAA) were performed [23].

### Definition of metabolic syndrome

We defined metabolic syndrome using the modified 2005 National Cholesterol Education Program Adult Treatment Panel III (NCEP-ATP III) definition as 3 or more of the following: waist circumference ≥40 inches in men or ≥35 inches in women; systolic blood pressure ≥130 or diastolic blood pressure ≥85 mm Hg or antihypertensive treatment in a patients with history of hypertension; triglycerides ≥150 mg/dL; HDL cholesterol ≤40 mg/dL in men or 50 mg/dL in women; and glucose ≥100 mg/dL or history of diabetes mellitus or use of anti-diabetic medication [24]. In addition, we categorized body-mass index (BMI) according to the World Health Organization (WHO) criteria: BMI <25 kg/m^2^  =  healthy weight, BMI 25–29.9 kg/m^2^  =  overweight, and BMI ≥30 kg/m^2^  =  obese.

### Statistical analysis

For analysis comparing cases and controls, continuous variables are shown as means (standard deviation) or medians (inter quartile range), and the unpaired t test or the Wilcoxon unpaired rank sum test compared cases and controls. Proportions were compared using the χ^2^ test. Spearman correlations assessed the relation between continuous variables. We used unconditional logistic regression to calculate the odds ratio and 95% confidence interval (CI) of coronary heart disease (CHD). Tests for linear trend, for both the linear regression and logistic regression models, used the median score variables. A probability value of 0.05 was considered statistically significant.

## Results

### Plasma PTX3 levels associate with features typical of metabolic syndrome, but do not predict recurrent coronary events

**Table 1** presents the mean values for selected characteristics among the controls, according to quartiles of plasma PTX3 concentration. Participants with higher PTX3 concentration generally had lower prevalence of metabolic syndrome, as well as favorable levels of various components associated with metabolic syndrome — including lower BMI, waist circumference, triglycerides, apoCIII and tPA, and higher levels of high-density lipoprotein (HDL) cholesterol. Participants with higher PTX3 concentrations also had higher levels of the inflammatory marker SAA, but not of CRP. High PTX3 concentration was not associated with fasting glucose, insulin or the HOMA index.

**Table 1:** Baseline characteristics of the 366 controls in the CARE populations by quartiles of PTX3.

	Quartile of PTX3				
	Q1	Q2	Q3	Q4	
Median PTX3	2.1	2.9	4.1	6.1	
Range	<2.6	2.6–3.4	3.5–5.0	>5.0	p-trend
Age (years)	56	59	60	62	<0.001
Male (%)	85	92	89	81	0.18
Current smoker (%)	12	8	8	13	0.76
History of diabetes (%)	4	12	7	13	0.15
History of hypertension (%)	41	34	40	46	0.29
Family history of CVD (%)	44	33	35	53	0.06
Body-mass index (kg/m^2^)	27.6	28.8	27.6	25.6	<0.001
Waist circumference (in)	38	40	38	36	<0.001
Metabolic syndrome (%)	46	48	46	32	0.03
Number of metabolic syndrome components	2.3	2.3	2.2	1.8	0.01
Total cholesterol (mmol/L)	190	183	193	193	0.20
HDL cholesterol (mmol/L)	40	40	42	45	<0.001
LDL cholesterol (mmol/L)	118	114	120	121	0.20
Triglycerides (mmol/L)	163	147	158	134	0.01
High-sensitivity CRP (mg/L)	3.8	4.4	4.9	5.3	0.13
Serum amyloid A (mg/L)	3.5	7.1	5.9	10.9	0.04
Apo CIII (mg/dL)	11.6	11.4	11.7	10.6	0.05
tPA, median (IQR) (ng/mL)	12.5	10.7	10.1	9.3	0.03
Fasting plasma glucose (mmol/L)	98	107	103	98	0.35
Fasting insulin (pmol/L)	8.6	10.8	8.8	10.9	0.65
HOMA-IR	2.2	2.9	2.4	2.7	0.78
Serum creatinine	1.14	1.12	1.07	1.13	0.67
MDRD-GFR	69.7	73.4	75.9	70.5	0.96
Medication use (%)					
Anti-coagulant	3	1	3	7	0.08
Anti-arrhythmic	50	54	43	42	0.17
Aspirin	73	84	87	75	0.94
Diabetic medications	3	7	4	6	0.52
Cholesterol medication	33	45	37	41	0.62
Hypertension medication	26	27	23	29	0.79

IQR  =  interquartile range. MDRD-GFR  =  Modification of Diet in Renal Disease-Glomerular Filtration Rate.

Compared with controls, cases had higher BMI and waist circumference, and more smoked cigarettes currently, had diabetes and hypertension, and took cardiovascular medications (**Table 2**). The cases had higher levels of total cholesterol, LDL cholesterol, triglycerides, and plasma glucose, and lower HDL cholesterol levels than the controls. No significant difference in plasma PTX3 levels occurred between the cases and controls. PTX3 did not predict CHD risk (nonfatal MI and fatal CHD), even after adjustment for metabolic syndrome components, other coronary risk factors, and medication use (**Table 3**).

**Table 2:** Characteristics of cases and controls within the CARE trial.

	Controls (n = 336)	Cases (n = 413)	*P*-value
Age (years)	60 (10)	60 (10)	0.73
Pentraxin, mean (min, max) (ng/ml)	3.9 (0.8, 12.5)	4.1 (0.5, 16.3)	
Pentraxin, median (IQR) (ng/ml)	3.5 (2.6, 5.1)	3.8 (2.7, 5.0)	0.12
Male (%)	87	87	0.83
Current smoker (%)	10	23	<0.001
History of diabetes (%)	9	22	<0.001
History of hypertension (%)	40	48	0.03
Family history of CVD (%)	41	38	0.29
Body-mass index (kg/m^2^)	27.4 (4.2)	28.3 (4.9)	0.01
Waist circumference (in)	38.1 (4.5)	39.0 (5.2)	0.01
Metabolic syndrome (%)	43%	56%	<0.001
Number of metabolic syndrome components	2.1 (1.3)	2.4 (1.3)	<0.001
Total cholesterol (mmol/L)	4.91 (0.80)	5.04 (0.80)	0.01
HDL cholesterol (mmol/L)	1.09 (0.25)	1.01 (0.25)	0.001
LDL cholesterol (mmol/L)	3.05 (0.70)	3.18 (0.72)	0.01
Triglycerides, median (IQR) (mmol/L)	1.52 (1.15, 2.07)	1.70 (1.25, 2.21)	0.003
High-sensitivity CRP (mg/L)	4.6 (7.0)	5.5 (7.3)	0.06
Serum amyloid A (mg/L)	6.9 (21.4)	7.2 (16.8)	0.79
Apo CIII (mg/dL)	11.3 (3.3)	12.1 (3.6)	0.003
tPA, median (IQR) (ng/mL)	9.6 (7.2, 12.0)	10.1 (7.5, 13.2)	0.10
Fasting plasma glucose (mmol/L)	5.66 (1.34)	6.16 (2.10)	<0.001
Fasting insulin, median (IQR) (pmol/L)	42.6 (31.2, 61.2)	42.6 (30.6, 55.8)	0.12
Serum creatinine	1.11 (0.2)	1.15 (0.2)	0.01
MDRD-GFR	72.4 (15)	70.2 (15)	0.04
Treatment group (%)	48	42	0.1
Medication use (%)			
Anti-arrhythmic	47	59	0.001
Anti-coagulants	4	7	0.04
Aspirin	80	78	0.59
Lipid-lowering drugs	9	13	0.05
Beta-blockers	39	46	0.04
Calcium channel blockers	36	40	0.21
Insulin	1	4	0.01
Oral hypoglycemic drugs	4	14	<0.001

All values are means (standard deviations) unless otherwise noted.

IQR  =  interquartile range. MDRD-GFR  =  Modification of Diet in Renal Disease-Glomerular Filtration Rate. Continuous variables are shown as means (standard deviation) or medians (inter quartile range).

**Table 3:** Odds ratio of CHD (nonfatal MI and fatal CHD) by quartiles of pentraxin 3 concentration.

	Quartiles of pentraxin				
	Q1	Q2	Q3	Q4	Trend
Range	0.83–2.60	2.60–3.48	3.48–5.04	5.05–12.5	
Median	2.14	2.92	4.15	6.08	
Cases	87	90	136	100	
Controls	91	92	91	92	
Age and sex adjusted	1.0 (ref)	1.03 (0.68–1.56)	1.59 (1.07–2.38)	1.17 (0.77–1.78)	0.29
Multivariate adjusted 1	1.0 (ref)	0.93 (0.60–1.44)	1.48 (0.97–2.25)	1.03 (0.66–1.59)	0.60
Multivariate adjusted 2	1.0 (ref)	0.96 (0.62–1.51)	1.55 (1.00–2.42)	1.08 (0.68–1.72)	0.48

Multivariate 1: adjusted for age, sex, smoking, left ventricular ejection fraction, and history of diabetes.

Multivariate 2: adjusted for age, sex, smoking, left ventricular ejection fraction, history of diabetes, CRP, SAA, metabolic risk factors (systolic blood pressure, diastolic blood pressure, glucose, HDL, triglycerides, waist circumference), glomerular filtration rate, and medication use (diabetic medication, hypertension medication, cholesterol medication, anti-arrhythmics, and anti-coagulants).

### PTX3 levels associate with components of metabolic syndrome

**Figure 1:**
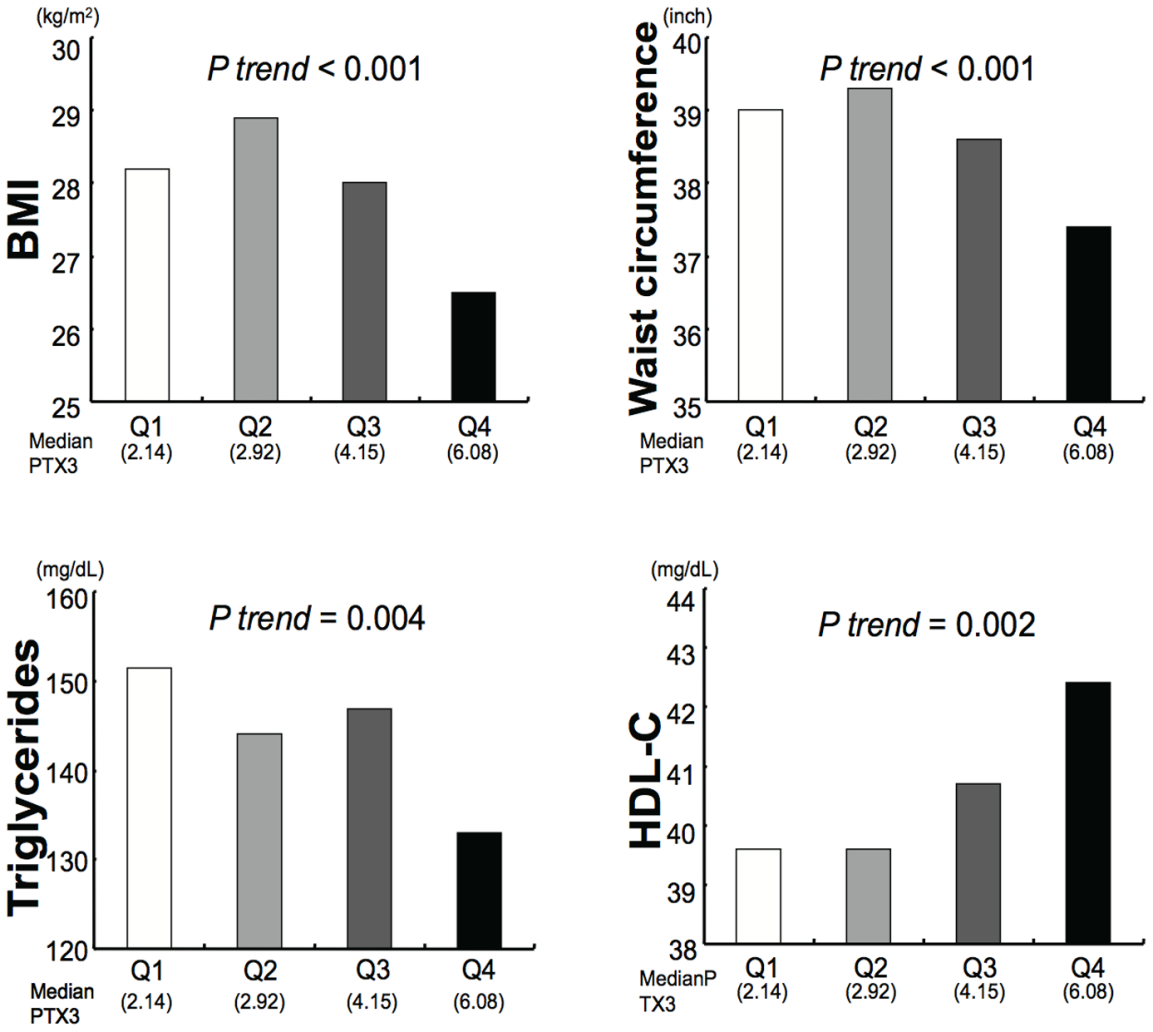
PTX3 levels associate adversely with metabolic syndrome components. Mean values of selected characteristics (BMI, waist circumference, triglyceride, and HDL cholesterol) by quartile of PTX3. Mean values were estimated from linear regression models, adjusted for age.

Individuals with metabolic syndrome had lower PTX3 levels (mean  = 3.89 ng/mL, SE  = 0.10, n = 397) than those without metabolic syndrome (4.22 ng/mL, SE  = 0.10, *P* = 0.02, n = 382), after adjustment for age, sex, and smoking. Additionally, plasma PTX3 levels correlated inversely with variables associated with metabolic syndrome (**Figure 1**), including BMI, waist circumference, and triglycerides, while positively correlating with HDL cholesterol after adjustment for age. Plasma PTX3 remained statistically significant in association with BMI (*P trend* <0.001) and waist circumference (*P trend* <0.001) even after adjustment for age, sex, smoking, history of hypertension, triglycerides, HDL cholesterol, and insulin.

### Lower PTX3 levels in obese, non-diabetic individuals

**Figure 2:**
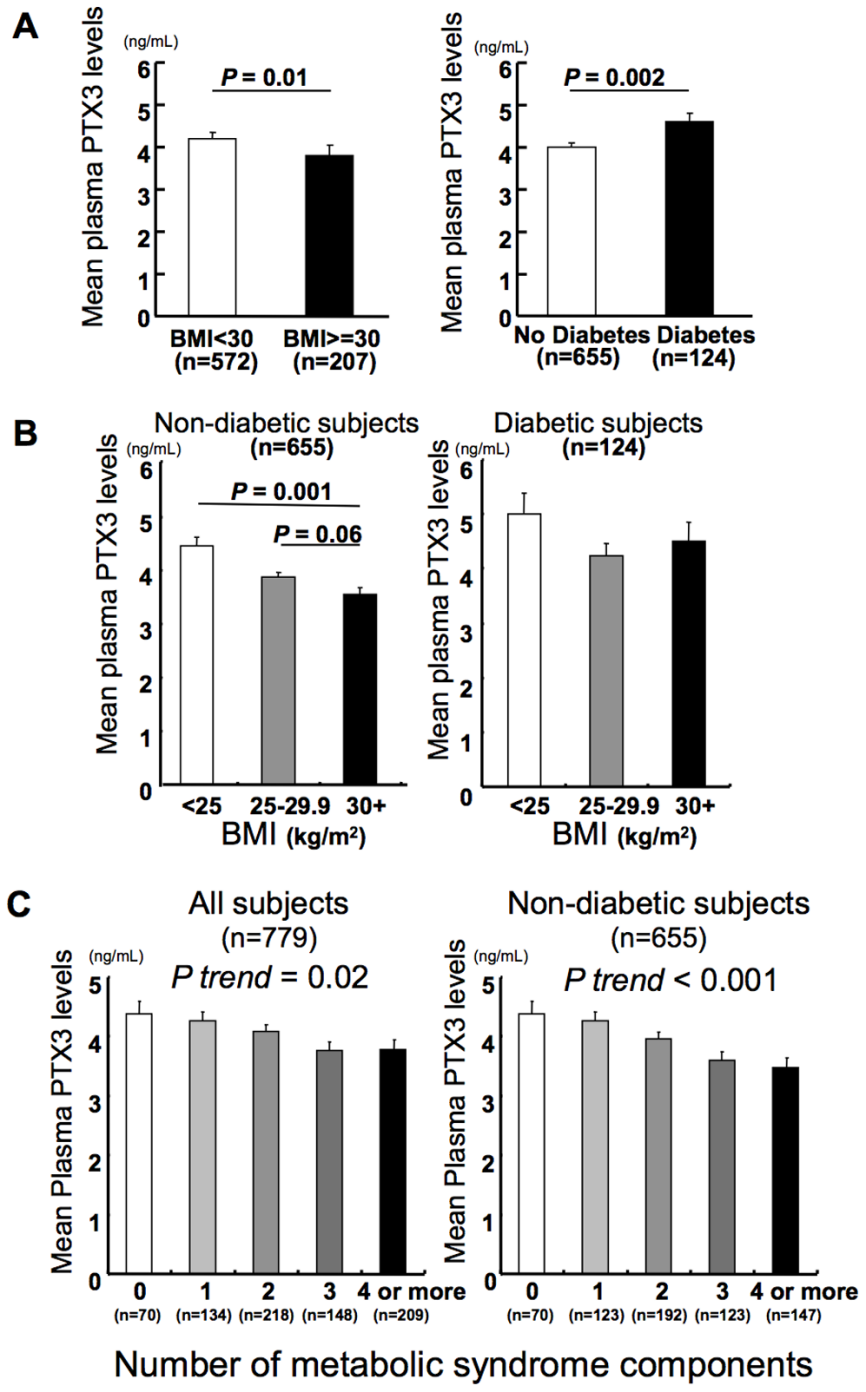
PTX3 associates negatively with obesity and metabolic syndrome in non-diabetic subject. PTX3 associates negatively with obesity but positively with diabetes mellitus (A). Mean plasma PTX3 levels among 779 individuals, comparing non-obese (BMI <30) and obese individuals (BMI ≥30) (left), or non-diabetic and diabetic control individuals (right). Mean values were estimated from linear regression models, adjusted for age, sex, smoking status, triglycerides, HDL cholesterol, and hypertension. Models for diabetes also adjusted for BMI. PTX3 negatively associates with obesity among non-diabetic individuals (B). Mean values of plasma PTX3 levels by BMI category in non-diabetic (left) and diabetic individuals (right) estimated from linear regression models. Mean values adjusted for age, sex, smoking status, triglycerides, HDL cholesterol, and hypertension. PTX3 associates negatively with the number of metabolic syndrome components (C). Mean values of plasma PTX3 levels according to presence of 0, 1, 2, 3, and 4 or more components of metabolic syndrome in all individuals (left) and non-diabetic individuals (right). Mean values estimated from linear regression models, adjusted for age, sex, and smoking status.

Obese patients had lower levels of adjusted mean PTX3 (mean  = 3.77 ng/mL, SE = 0.13) than did non-obese patients (mean  = 4.15 ng/mL, SE = 0.08, **Figure 2A**). Conversely, patients with diabetes (mean  = 4.53 ng/mL, SE  = 0.11) had higher adjusted mean PTX3 concentrations than did controls without diabetes (mean  = 3.96 ng/mL, SE  = 0.07, **Figure 2A**). To probe further the association of PTX3 and obesity, we stratified the control subjects by diabetic status. Compared to patients with a healthy weight (BMI <25), both overweight patients (25≤ BMI <30) and obese patients (BMI ≥30) had lower mean plasma PTX3 levels among those without diabetes (n = 655), after adjustment for confounders (**Figure 2B**). Patients with diabetes (n = 124) had no significant difference in mean plasma PTX3 levels in different categories of BMI (**Figure 2B**). Of the 124 patients with diabetes mellitus, 88 patients (71%) took medications for this condition.

### PTX3 levels associate inversely with components of metabolic syndrome in all individuals and non-diabetic individuals

We further explored the association between PTX3, metabolic syndrome, and diabetes mellitus. PTX3 associated inversely with the total number of metabolic syndrome components in all individuals (n = 779) after adjustment for age, sex, and smoking (**Figure 2C**). Non-diabetic patients (n = 655) showed a stronger inverse association between PTX3 and total number of metabolic syndrome components (**Figure 2C**).

### PTX3 levels do not correlate with CRP levels

**Figure 3:**
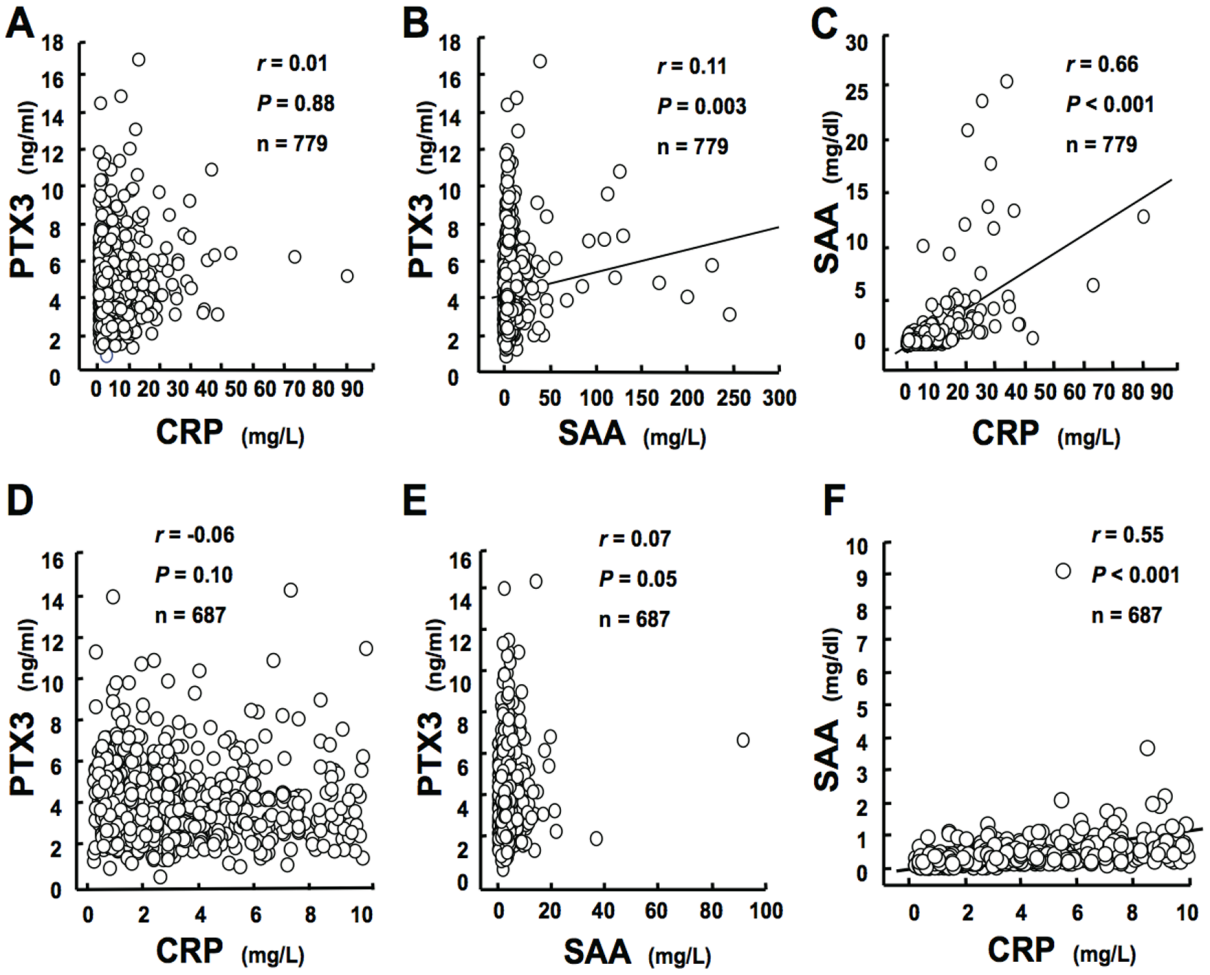
Lack of association between PTX3 and short pentraxin. Spearman correlations revealed no association between plasma levels of the long pentraxin PTX3 and the short pentraxin CRP (A). Plasma PTX3 levels positively correlated with plasma SAA levels (B), and plasma CRP and SAA levels correlated strongly with each other (C). After exclusion of participants with high CRP levels (>10 mg/L), PTX3 showed no significant correlations with CRP and SAA (D and E), whereas CRP and SAA correlated positively with each other (F).

PTX3 levels did not correlate with levels of the short pentraxin CRP (**Figure 3A**), and very weakly and positively correlated with another inflammatory marker, SAA (**Figure 3B**). CRP and SAA correlated with each other strongly and positively (**Figure 3C**). After exclusion of participants with CRP above 10 mg/L (n = 687), PTX3 showed no significant correlations with CRP or SAA (**Figure 3D and 3E**), whereas CRP and SAA correlated positively with each other (**Figure 3F**), suggesting that different mechanisms regulate levels of the long and short pentraxin in these MI survivors.

## Discussion

This analysis from the CARE trial tested the hypothesis that plasma levels of the immunomodulator PTX3 predict risk of acute complications of coronary atherosclerosis in a population of MI survivors and also associated with cardiometabolic disorders. Although our results demonstrate that plasma PTX3 levels associate inversely with components of metabolic syndrome, the study does not support a relation between this pentraxin and recurrent CHD risk, unlike other pentraxin family members such as CRP.

Several clinical studies have reported elevated plasma PTX3 levels in patients following acute MI or unstable angina [5]–[7]. In patients with heart failure, elevated plasma PTX3 levels associated independently with cardiac events but did not specifically correlate with coronary events [8], [14]. Other clinical studies indicated that PTX3 levels associate with cardiovascular disease and all-cause death among apparently healthy older individuals and patients with CHD [11], [16]. These studies led to our intriguing hypothesis that PTX3 serves as a predictor of cardiovascular risk. In the population studied here with prior history of MI, however, plasma PTX3 levels did not predict subsequent events. The difference in the study population (e.g., primary vs. secondary prevention) may pertain to this discrepancy, although other risk factors including CRP have similar associations with cardiovascular disease in primary and secondary prevention. The pro-inflammatory or anti-inflammatory role of PTX3 may also depend on the relative contribution of producing cell types, the disease context, and/or the stage of disease. Although PTX3 may associate with coronary events in other populations, the present study does not support its role as a risk predictor.

The causal role of PTX3 in the pathogenesis of atherosclerosis remains obscure. Macrophages, endothelial cells, smooth muscle cells, and neutrophils abundantly express PTX3 in human atherosclerotic lesions [25], [26]. *In vitro* studies demonstrated that pro-inflammatory stimuli (e.g., LPS, TNF-α, IL-1β, and oxidized LDL) [27]–[30] and anti-inflammatory stimuli (e.g., IL-10 and HDL) [31], [32] enhance PTX3 production. In rats, forced expression of PTX3 reduces neointima formation after balloon injury, indicating that this long pentraxin can limit intimal hyperplasia [9]. PTX3-deficient mice with acute MI showed increased myocardial damage, and exogenous PTX3 expression rescued this phenotype, suggesting a cardioprotective function of PTX3 [10]. Furthermore, PTX3 deficiency in mice worsened atherosclerotic lesions and inflammatory profiles [12]. Further investigations may elucidate the specific role of PTX3 in various contexts of cardiovascular disease.

Obesity and metabolic syndrome represent chronic inflammatory states [33]–[35]. Whereas plasma levels of the short pentraxin CRP are higher in obese individuals and those with metabolic syndrome [36], evidence linking PTX3 with obesity and metabolic syndrome remains scant. The present study using MI survivors, and another clinical study in a healthy Japanese population, found that PTX3 is inversely correlated with obesity and metabolic syndrome [20]. In addition, patients with end-stage renal disease show inverse correlations of plasma PTX3 levels with BMI and waist circumference [15]. Another study reported decreased PTX3 mRNA expression in mature adipocytes derived from human adipose tissue, compared to that in preadipocytes [37]. These divergent results for CRP and PTX3 results may reflect different sources and biologic roles of the short and long pentraxins. Previous studies, including our own, have reported that various cell types associated with atherosclerosis and metabolic syndrome produce PTX3 in response to inflammatory signals, while the liver elaborates most circulating CRP [1], [27], [29], [30], [38], [39]. HDL induces PTX3 mRNA expression and protein release in human endothelial cells through activation of the PI3K/Akt pathway [32], which may contribute to low plasma PTX3 levels in individuals with metabolic syndrome who have low HDL levels. PTX3 correlated inversely with triglycerides and with apoCIII, which interferes with clearance of triglyceride-rich lipoproteins from plasma [40]. We recently established that apoCIII induces cell activation and inflammation in vitro and in vivo [41]. Interaction between apoCIII and PTX3 requires further study. While several previous reports demonstrated significant positive associations between PTX3 and CRP [42], others showed no significant correlation [5], [6]. PTX3 did not correlate significantly with CRP in our population. The distinct behaviors of PTX3 compared with those of CRP also suggest that long and short pentraxins have different relationships with the pathogenesis of obesity and metabolic syndrome.

In this study population, individuals with diabetes had higher plasma PTX3 levels than non-diabetic individuals although PTX3 did not correlate with fasting glucose, insulin or the HOMA-IR. More than 70% of diabetic patients took medications for diabetes mellitus, suggesting that these treatments affected the association between plasma PTX3 levels and diabetes mellitus in our study population. To clarify the effect of obesity on plasma PTX3 levels independent of diabetes mellitus, we divided study participants into diabetic and non-diabetic groups. Among individuals without diabetes, obese individuals had lower plasma PTX3 levels than lean individuals. Moreover, non-diabetic individuals showed a significant inverse correlation between PTX3 and the number of metabolic syndrome components.

This study has a few limitations. We did not measure PTX3 levels in patients who received pravastatin. The present study tested the specific hypothesis that plasma PTX3 levels predict risk of recurrent cardiovascular events in MI survivors. We thus measured PTX3 levels only at the baseline in case and control subjects. Our results indicated that PTX3 levels did not associate with future recurrent events in the CARE study cohort. As the present study did not to identify PTX3 as a biomarker of cardiovascular risk, we did not measure PTX3 levels during the follow-up period in control and pravastatin-treated subjects. Several studies have linked higher PTX3 levels with heart failure [8], [14]. Because CARE did not adjudicate heart failure endpoints, we did not examine the association between PTX3 levels and heart failure.

The present study indicates that PTX3 does not predict future cardiac events, unlike CRP, although it associates with reduced levels of cardiometabolic risk factors. Various features associated with metabolic syndrome correlate negatively with PTX3 levels but positively with CRP. The underlying mechanisms may involve complex processes regulating PTX3 production in various cell types activated in the pro-inflammatory microenvironment of patients with cardiometabolic syndrome. Although the present study offers clinical evidence that further links PTX3 with metabolic syndrome, understanding the biology of this immunomodulator in cardiometabolic disorders requires additional multidisciplinary investigations.

## Supporting Information

Methods S1 **Supplemental data for detailed methods.** Supplemental information of study population, blood collection and laboratory measurements, definition of the metabolic syndrome, and statistical analysis.
(DOC)
