# Supplementary material for: Plasma Pentraxin 3 Levels Do Not Predict Coronary Events but Reflect Metabolic Disorders in Patients with Coronary Artery Disease in the CARE Trial
Source: PLoS One. 2014 Apr 4;9(4):e94073. doi: 10.1371/journal.pone.0094073 (PMC3976379; doi:10.1371/journal.pone.0094073)
Supplement: Methods S1 — Supplemental data for detailed methods. Supplemental information of study population, blood collection and laboratory measurements, definition of the metabolic syndrome, and statistical analysis. (DOC) [file pone.0094073.s001.doc]

Miyazaki et al. Plasma pentraxin 3 levels do not predict coronary events but reflect metabolic disorders in patients with coronary artery disease in the CARE trial.

**Supplemental Methods**

**Study population**

We conducted a prospective, nested, case-control study within the CARE trial. The CARE trial, a randomized, double-blind, placebo-controlled trial, included 4159 patients (86% men) residing in the United States or Canada with a prior myocardial infarction (MI) that occurred between 3 and 20 month before enrollment who had total cholesterol levels < 240 mg/dL and LDL cholesterol levels between 115 and 175 mg/dL. Patients were randomized to 40 mg pravastatin or placebo for a median of 5 years. The institutional review board at each participating clinical center approved the study, and individuals gave written informed consent. A complete list of the staff of the trial organization and the clinical and coordinating center has been described previously. During the trial, 486 patients experienced the primary endpoint of coronary death or MI. Sufficient plasma for analysis was available from both screening visits for 413 of these cases. We randomly selected control individuals from patients who did not have a primary endpoint, matched to the cases on decade of age (e.g., 40 to 49, 50 to 59 years) and sex. Among the 419 matches, we excluded 53 patients who had coronary bypass surgery, coronary angioplasty, or stroke after randomization during follow-up. Measurement of PTX3 levels of these blood samples from the CARE trial and this analysis were approved by the committee for the Protection Against Research Risks of Brigham and Women’s Hospital, Harvard medical school.

**Blood collection and laboratory measurements**

A fasting blood sample was taken from each patient on each of 2 screening visits
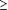
 1 week apart and sent by overnight delivery in cooled containers to the core laboratory in St. Louis, Missouri. EDTA served as an anticoagulant and preservative. Plasma was separated in a refrigerated centrifuge, and 1-mL aliquots were placed in polypropylene vials and stored continuously at-80°C until analysis. PTX3 was measured by a high-sensitivity ELISA (Perseus Proteomics Inc, Tokyo, Japan) as previously described. The coefficient of variations was less than 11% for blinded split samples obtained from a plasma pool from healthy volunteers. Plasma total cholesterol, high-density lipoprotein (HDL) and low-density lipoprotein (LDL) cholesterol, triglycerides, fasting blood glucose, apolipoproteins CIII (apoCIII), tissue plasminogen activator (tPA) and serum creatinine were measured as previously described. High sensitivity assays for CRP and SAA were performed according to methods described previously. We used the Modification of Diet in Renal Disease glomerular filtration rate (MDRD-GFR) formula to estimate renal function. Plasma insulin levels were measured by a commercially available ELISA kit (Alpco Diagnostics, Salem, NH). Homeostasis model assessment of insulin resistance (HOMA-IR) was calculated from fasting glucose and insulin concentrations. All personnel at the laboratories were blinded to the case-control status, which was maintained at the Data Coordinating Center, University of Texas School of Public Health, Houston.

**Definition of the metabolic syndrome**

We defined metabolic syndrome using the modified 2005 NCEP-ATP III definition as 3 or more of the following: waist circumference ≥40 inches in men or ≥35 inches in women; systolic blood pressure ≥130 or diastolic blood pressure ≥85 mm Hg or antihypertensive treatment in a patients with history of hypertension; triglycerides ≥150 mg/dL; HDL cholesterol ≤40 mg/dL in men or 50 mg/dL in women; and glucose ≥100 mg/dL or history of diabetes mellitus or use of anti-diabetic medication. In addition, we categorized body mass index (BMI) according to the WHO criteria: BMI <25 kg/m^2^ = healthy weight, BMI 25–29.9 kg/m^2^ = overweight, and BMI ≥30 kg/m^2^ = obese.

**Statistical analysis**

For analysis comparing cases and controls, continuous variables are shown as means (standard deviation) or medians (inter quartile range), and the unpaired t test or the Wilcoxon unpaired rank sum test compared cases and controls. Proportions were compared using the χ^2^ test. Spearman correlations assessed the relation between continuous variables. We calculated the means and proportions for baseline characteristics across quartiles of PTX3. To test the significance of association across quartiles, we assigned the median value of PTX3 to each quartile (a “median score” variable) and modeled this variable as a continuous variable using linear regression models for continuous variables and generalized estimating equations for categorical variables. To predict mean concentration of PTX3 within categories of metabolic and cardiovascular risk factors, we used multivariate linear regression to calculate least-squares mean and standard errors, adjusting for potential confounders. Additionally, we used multivariate linear regression to calculate least-squares mean and standard errors to estimate components of the metabolic syndrome within quartiles of PTX3. Triglycerides, insulin, and HOMA-IR were not normally distributed, thus these variables were log-transformed in the linear regression model and geometric means were estimated.

We used unconditional logistic regression to calculate the odds ratio and 95% confidence interval (CI) of coronary heart disease (CHD). Participants were categorized into quartiles based on the distribution of PTX3 levels in the control participants. Models were adjusted for various coronary risk factors and for medication use. Tests for linear trend for both the linear regression and logistic regression models were using the median score variables. A probability value of 0.05 (2-sided) was considered significant.

**References**

1. Sacks FM, Pfeffer MA, Moye LA, Rouleau JL, Rutherford JD, et al. (1996) The effect of pravastatin on coronary events after myocardial infarction in patients with average cholesterol levels. Cholesterol and Recurrent Events Trial investigators. N Engl J Med 335: 1001-1009.

2. Pfeffer MA, Sacks FM, Moye LA, Brown L, Rouleau JL, et al. (1995) Cholesterol and Recurrent Events: a secondary prevention trial for normolipidemic patients. CARE Investigators. Am J Cardiol 76: 98C-106C.

3. Inoue K, Sugiyama A, Reid PC, Ito Y, Miyauchi K, et al. (2007) Establishment of a high sensitivity plasma assay for human pentraxin3 as a marker for unstable angina pectoris. Arterioscler Thromb Vasc Biol 27: 161-167.

4. Sacks FM, Alaupovic P, Moye LA, Cole TG, Sussex B, et al. (2000) VLDL, apolipoproteins B, CIII, and E, and risk of recurrent coronary events in the Cholesterol and Recurrent Events (CARE) trial. Circulation 102: 1886-1892.

5. Ridker PM, Rifai N, Pfeffer MA, Sacks F, Braunwald E (1999) Long-term effects of pravastatin on plasma concentration of C-reactive protein. The Cholesterol and Recurrent Events (CARE) Investigators. Circulation 100: 230-235.

6. Tonelli M, Moye L, Sacks FM, Kiberd B, Curhan G (2003) Pravastatin for secondary prevention of cardiovascular events in persons with mild chronic renal insufficiency. Ann Intern Med 138: 98-104.

7. Froissart M, Rossert J, Jacquot C, Paillard M, Houillier P (2005) Predictive performance of the modification of diet in renal disease and Cockcroft-Gault equations for estimating renal function. J Am Soc Nephrol 16: 763-773.

8. Grundy SM, Cleeman JI, Daniels SR, Donato KA, Eckel RH, et al. (2005) Diagnosis and management of the metabolic syndrome: an American Heart Association/National Heart, Lung, and Blood Institute Scientific Statement. Circulation 112: 2735-2752.
